# False Dogmas in Schizophrenia Research: Toward the Reification of Pathway Phenotypes and Pathway Classes

**DOI:** 10.3389/fpsyt.2021.663985

**Published:** 2021-06-17

**Authors:** Michael Maes, George Anderson

**Affiliations:** ^1^Department of Psychiatry, Faculty of Medicine, Chulalongkorn University, Bangkok, Thailand; ^2^Department of Psychiatry, Medical University of Plovdiv, Plovdiv, Bulgaria; ^3^The Institute for Mental and Physical Health and Clinical Translation Strategic Research Center, Barwon Health, Deakin University, Geelong, VIC, Australia; ^4^Clinical Research Communications Centre, London and Scotland, London, United Kingdom

**Keywords:** schizophrenia, neuroimmune, inflammation, oxidative and nitrosative stress pathways, bacterial translocation

## Introduction

Conceptualizations of schizophrenia have always been controversial and subject to considerable argument. Many views prevail as to the nature of schizophrenia, including schizophrenia as: a brain disorder; a unitary disease, via diagnosis derived from DSM (American Psychiatric Association, APA) or ICD (World Health Association, WHO) classification systems; being comprised of positive and negative symptoms; an incurable brain disease, with a progressive deterioration that is determined by a dementing or neuroprogressive course. All of these perspectives have been challenged at some time, and none are universally accepted as descriptive of the processes underpinning schizophrenia phenotypes. Our work indicates that none of these conceptualizations of schizophrenia are supported by recent findings, which are detailed below and in Table 1. Our work showed that these false conceptualizations of schizophrenia had a negative impact on the development of more accurate diagnostic criteria, new pathways and new drug targets of schizophrenia phenotypes.

**Table 1:** False dogmas in schizophrenia research and the way forward.

**False dogmas**	**New findings**	**The way forward**
1. Schizophrenia is a brain disorder	No, it is a systemic disorder with peripheral IO&NS pathways causing the central phenome of schizophrenia.	Measurement of peripheral IO&NS pathways affecting neuronal circuits, as indicated by cognitive deficits in memory and executive functions and (functional) brain imaging techniques.
2. Schizophrenia is a unitary disease	No, there are distinct phenotypes, including deficit vs. non-deficit, partial vs. non-responders to treatment, FES and MES with and without worsening.	Stop publishing findings based on “schizophrenia,” as the findings using these case definitions are difficult to interpret.Consider the different pathway phenotypes.
3. The DSM/ICD case definitions of schizophrenia are the gold-standard to diagnose schizophrenia.	No, those case definitions are not only unreliable, but their dogma-like nature also prevents deductive and inductive remodeling of the case-definition.	The DSM/ICD committees should stop making ex-consensus diagnostic criteria and should consider pathway phenotypes and classes cross-validated using machine learning and the nomothetic network approach (1).
4. Schizophrenia is comprised of positive and negative symptoms	No, the symptomatome comprises many more symptom domains and a single latent trait, which reflects severity of the phenotypes, underpins these different manifestations and therefore is the cause of its manifestations.	Consider the different domains in the phenome of the schizophrenia phenotypes, namely psychosis, hostility, excitation, mannerism, negative symptoms, psychomotor retardation, formal thought disorders, affective symptoms, fatigue and physiosomatic symptoms. Extract the first latent vector, which reflects OSOS.
5a. Schizophrenia is accompanied by distinct cognitive impairments.	No, a general factor, which is essentially unidimensional, underpins all those different cognitive impairments.	Compute a general cognitive decline (G-CoDe) score as the first latent vector extracted from different cognitive scores.
5b. Patients with schizophrenia may show a gradual deterioration which may be assessed by a decline in cognitive scores.	No, this deterioration should be defined based on worsening in behavioral, cognitive, physical, and psychosocial domains.	Compute a behavioral-cognitive-physical-psychosocial (BCPS)-worsening score defined as a latent vector extracted from OSOS, G-CoDe, and health-related quality of life assessments, thereby combining all phenome (symptomatome and phenomenome) feature sets into one worsening index.
5c. Schizophrenia is a dementing or neuroprogressive disorder.	No, the findings show a far more complex picture. A serious deficit can be present following a first episode psychosis. FES/MES may present with or without worsening. The worsening in FES/MES, is associated with IO&NS pathways, and severe worsening largely overlaps with the deficit syndrome in MES or FES.	Consider staging pathway phenotypes, namely FES and MES with and without BCPS worsening.
5d. Many patients show complete remission as assessed with remitter case definitions based on scale-derived cut-off values or a number of items on a rating scale score being rated mild or better.	Probably not. Using the adequate machine learning techniques, not one of the partial responders to treatment could be allocated to the normal control class modeled using pathway phenotypes. These partial remitters show residual psychotic symptoms, neurocognitive deficits and active IO&NS pathways.	Complete remission, partial remission and non-remission should be modeled using SIMCA with pathway phenotypes (IO&NS pathways combined with neurocognitive tests) as modeling and discriminatory variables. The case definition of “complete remission” should be based on a SIMCA model, namely authentication of cases as belonging to the model of normal controls constructed using pathways and cognitive tests.
6. The way forward?	The novel bottom-up, data-driven, computer science-derived, nomothetic psychiatric approach as proposed by Maes et al. (2, 3) and Stoyanov and Maes (4).	This approach provides a route toward a novel model of schizophrenia based on all features sets, a new network-based definition of schizophrenia phenotypes and pathway classes. The latent variable scores delineate an idiomatic feature profile, which is unique for each patient and may be employed for individualized treatments targeting the most disordered feature sets of the nomothetic model.

*IO&NS, immune-inflammatory and oxidative and nitrosative stress; FES and MES, first and multiple episode schizophrenia; SIMCA, soft independent modeling of class analogy*.

### Schizophrenia: Not Only a Brain Disorder

A number of influential psychiatrists, including Nancy Andreasen, David Pickar, E. Fuller Torrey, and Robert Yolken, have helped to conceptualize schizophrenia as an incurable brain disease, thereby being similar to classically defined brain diseases, such as multiple sclerosis, Alzheimer's disease and Parkinson's disease. However, just as with such classical brain diseases (4–6), there is a growing realization that schizophrenia pathophysiology is more “holistic” in nature, being intimately linked to systemic processes, especially activation of immune-inflammatory, oxidative and nitrosative stress (IO&NS) pathways and increased gut permeability (2, 3, 7). Consequently, the early developmental pathoetiology of schizophrenia is associated with alterations in the regulation and interactions of systemic processes (8, 9). Furthermore, maternal immune activation as a consequence of bacterial and viral infections may cause schizophrenia-like symptoms in the offspring and these effects are mediated by IO&NS pathways and lowered neuroprotection (10). The first episode of psychosis (FEP) is typically accompanied by a cytokine storm indicative of immune-inflammatory response system (IRS) activation, coupled to an elevated compensatory immune-regulatory response system (CIRS), indicative of a “mixed” immune response (11). Such immune dysregulation may arise from viral/bacterial infections or complement activation (11). Typically, FEP shows a relatively increased IRS/CIRS activation ratio, as do many defined phenotypes including acute episodes, chronic schizophrenia, deficit schizophrenia, and treatment resistant schizophrenia (11, 12), with low-CIRS patients having poorer clinical outcomes (11, 12).

Consequences arising from the increased pro-inflammatory cytokines in FEP, include indoleamine 2,3-dioxygenase (IDO) induction, which drives tryptophan down the tryptophan catabolite (TRYCAT) pathway (13, 14). TRYCATs such as xanthurenic acid and picolinic acid, elevated pro-inflammatory cytokines/chemokines (e.g., CCL11), reactive oxygen and nitrogen species, and LPS (arising from breakdown of the paracellular and vascular gut barriers) are neurotoxic (2, 3). As such, alterations in neurotoxic activity may be intimately linked to changes in central and systemic neuronal and glia activity, thereby contributing to dysregulated neuronal patterning. Consequently, many of the cognitive deficits that can be associated with people classed with schizophrenia may be intimately linked to the interactions of systemic and central processes (2, 3).

### Schizophrenia: Not One Disease, but Distinct Phenotypes

Although regarded as a heterogeneous group of conditions (15, 16), the DSM and ICD classification systems consider schizophrenia as a unitary disease comprised of variable subgroups (such as paranoia, undifferentiated), with the subgroups coming and going across various DSM/ICD versions over time. The lack of diagnostic criteria based on biomarkers coupled with clinical features is a general problem for these classification systems (2, 4, 17).

One classical view is that negative symptoms are specific for schizophrenia and especially for deficit schizophrenia, although negative symptoms display a continuous distribution from healthy individuals to patients with deficit schizophrenia (18). Nevertheless, using soft independent modeling of class analogy (SIMCA), stable schizophrenia presentations could be divided into two qualitatively distinct phenotypes, namely deficit and non-deficit schizophrenia (14). SIMCA showed that deficit schizophrenia is defined by IO&NS pathways, neurocognitive deficits in episodic and semantic memory, increased severity of symptoms including psychosis, hostility, excitation, mannerism, negative symptoms (PHEMN), psychomotor retardation, and formal thought disorders, and a lowered quality of life (19). As well as such continuous variable differences, deficit schizophrenia is also associated with more qualitative differences, including lowered natural immunoglobulin (Ig)M responses to oxidative specific epitopes (OSEs), antioxidants and antibacterial defenses (2). Cognitive deficits may be combined with systemic pathway biomarkers into a neuro-immune brain-circuit axis phenotype, which may be employed as a tool for making the diagnosis of deficit schizophrenia (20).

SIMCA also shows that treatment partial responders, vs. non-responders, form qualitatively distinct classes when using cognitive and systemic pathways, with high accuracy (21). Partial, vs. non-responders may also be differentiated by increased IL-6, endogenous opioid system biomarkers and inhibition of the Wnt signaling pathway (21). Schizophrenia phenotypes may also be differentiated by affective symptoms, fatigue and physiosomatic (formerly psychosomatic) symptoms, all of which are associated with immune/cytokine/TRYCAT/O&NS/gut-brain pathways, clearly indicating the achievability of pathway-phenotype differentiation of people classed with schizophrenia phenotypes (22, 23).

### The Schizophrenia Case Definitions of DSM and ICD Are Counterproductive

DSM and ICD are also criticized for providing case definitions of schizophrenia that are based on descriptive psychopathology and de-contextualized narratives of the disorder (4), with problems arising from: (1) lumping qualitatively distinct classes (deficit vs. non-deficit; partial vs. non-responders to treatment) into one category; and (2) top-down determination prior to physiological and neurocognitive investigation (4), with any such measures seen as mere concomitant data. As a consequence of the poor pathway-phenotype basis to classification and treatment in people presenting with schizophrenia, DSM/ICD case definitions show poor reliability/validity, and little consistency across DSM/ICD updates (4).

It is incomprehensible that most biological and molecular research reports define these top-down DSM/ICD case definitions as independent variables, whilst using biomarkers and even causome, protectome and cognitome features as dependent variables. Consequently, while causal reasoning indicates that those features are explanatory variables and schizophrenia is a higher-order concept consisting of these features, researchers continue to use inadequate model assumptions, often confounded by the use of inappropriate statistical tests (3, 4). As such, an inappropriate statistical analysis arises from bestowing primacy to DSM/ICD classifications.

### Positive and Negative Symptoms: an Inappropriate Division

Another top-down dogma in psychiatry's classical conceptualization of schizophrenia is viewing such presentations as comprised of positive and negative symptoms, defined as the addition and loss of processes and behaviors, such as hallucinations and social isolation, respectively (15, 24–26). However, we have shown that a single latent unidimensional trait underpins such diverse presentations as PHEMN symptoms, psychomotor retardation, and formal thought disorders with good convergent validity, internal consistency reliability, and predictive relevance that follows a reflective model (27, 28). This unidimensional trait, or latent phenomenon, reflects overall severity of schizophrenia (OSOS). With increasing illness severity the quantitative differences in OSOS become more pronounced thereby shaping a qualitatively distinct class (19) especially in patients with a lowered CIRS protection (2).

### Neuroprogression or Total Recovery?

Kraepelin's “dementia praecox” has led to a long-standing belief that schizophrenia is a progressively deteriorating disorder. More recent terminology sees this as neuroprogression, indicative of a deterioration through a series of stages (29). However, some findings have always been incompatible with this, indicating that almost complete recovery may not uncommonly occur (30). Nevertheless, the concepts “cognitive decline,” “progressive deterioration” and “complete recovery” were never well-defined.

A classical view is that schizophrenia is accompanied by many cognitive impairments including in executive functions, semantic and episodic memory, attention, and spatial working memory (31). Nevertheless, we have shown that a common core underpins these neurocognitive deficits which should be denoted as “general cognitive decline” (G-CoDe). Our recent work indicates that “progressive deterioration” or “worsening” in schizophrenia phenotypes should be comprised of OSOS, G-CoDe, and psychosocial and general health domains (22). Pathways underpinning the worsening in first episode schizophrenia (FES) include elevations in indicants of paracellular gut and vascular barrier breakdown, with heightened levels of bacterial translocation (perhaps especially *Klebsiella pneumoniae*), complement C1q activation, and lowered antioxidant defenses (22). However, in multiple episode schizophrenia (MES), worsening is predicted by the number of episodes as well as heightened IO&NS pathways (22). The greatest deterioration largely occurs in people who would be classed with deficit schizophrenia. Such data would suggest that there is a readily measurable pathway substrate to the association of 'cognitive, social and general health domains' with worsening in people classed with schizophrenia that may strongly overlap with the biological underpinnings of deficit schizophrenia.

Classically, the case definitions of complete remission are based on scale-derived cut-off values or eight items of the PANNS being rated mild or better (32). Nevertheless, we showed that complete remission should be defined using SIMCA whereby a SIMCA model of healthy controls is constructed based on the neuro-immune biomarker values and cognitive scores of heathy controls (21). Consequently, using SIMCA, cases considered to be apparent responders to treatment may be projected into this SIMCA model and be allocated or not to this healthy control class (21). Cases that are allocated to the control class are “authenticated” as complete responders, whereas rejection to this normal class membership indicates that the patient did not achieve complete remission (21). Importantly, we found that using SIMCA none of the treatment partial responders could be authenticated as belonging to the SIMCA model of heathy controls (21). By inference, none of the treated schizophrenia patients could be considered as a complete remitter (21).

## Discussion

As indicated above, the classical dogmas of DSM/ICD classifications, including in the definition of schizophrenia, are widely regarded as inadequate.

1. Schizophrenia is clearly not simply a brain disease but a systemic neuro-immune and neuro-oxidative stress disorder just like other neurologic diseases, including multiple sclerosis, Parkinson's disease and Alzheimer's disease. Breakdown of paracellular and vascular pathways may lead to BBB breakdown thereby interfering with neuronal circuits which underpin the neurocognitive deficits and symptoms of schizophrenia phenotypes.
2. Schizophrenia is not one unitary disease but comprises different qualitatively distinct phenotypes including deficit and non-deficit schizophrenia, partial and non-responders to treatment, MES and FES with and without worsening. The failure to use such schizophrenia phenotypes complicates comparisons across studies, resulting in a plethora of “mixed results” that adds to the confusion and seemingly intractable nature of schizophrenia. Research based on DSM/ICD criteria suffers from false negative (not exposing phenotype-specific pathways) and false positive (a pathway being specific for only one phenotype is generalized to schizophrenia) results.
3. The top-down nature of DSM/ICD case definitions of schizophrenia are not only unreliable, but their dogma-like nature also prevents deductive (as incontrovertible) and inductive (as top-down) remodeling of the case-definition (4). Importantly, these models do not pass Karl Popper's critical rationalism tests, being non-progressive (not based on all available knowledge), unchangeable (ex consensus-based committees decide), and unfalsifiable (top-down manner precludes refutation) (4). The utilization of DSM/ICD criteria is inhibiting a pathway-based understanding and treatment of schizophrenia phenotypes.
4. The classical bidimensional concept of positive and negative symptoms is another dogma that is not supported by the findings. This is intimately intertwined with counterproductive debates as to the dimensional vs. categorical (distinct phenotypes) conceptualizations of schizophrenia (14, 19). “Schizophrenia” comprises different subtypes which are modeled by pathway phenotypes, which increase in severity along a continuum and give rise to qualitatively differences among those phenotypes (2, 14, 19).
5. Conceptualizing schizophrenia-like presentations as having a neuroprogressive or dementing course seems inadequate. A serious deficit can be present following FES and FES/MES may present with no or minimal deterioration (22). Complete remission should be modeled using SIMCA rather than with case definitions based on scale-derived cut-off values or eight items of the PANNS being rated mild or better (32). Based on SIMCA results, we conclude that complete recovery is probably never achieved, as patients show residual psychotic symptoms, neurocognitive deficits and active immune-inflammatory pathways (21). Our findings extend Bleueler's view that patients may return to a normal functioning, albeit with scarring. Of clinical note, patients showing a partial treatment response, irrespective of first or multiple episode(s), still show heightened activation of treatable pathophysiological processes, the recognition and treatment of which may improve the condition.

### The Way Forward: Bottom-Up Nomothetic Networks

Psychiatry can only make progress when the gold-standard top-down approach is abandoned and is replaced by the novel bottom-up, data-driven, computer science-derived, nomothetic psychiatric approach (2–4). This approach first builds a theoretical framework which is based on state-of-the-art knowledge and causal reasoning and assembles the building blocks (feature sets) of schizophrenia into one framework, comprising the causome and protectome and their disbalance computed as a risk to resilience ratio (2, 3), the adverse outcome pathways, namely the different pathways that may cause the illness, the brainome (including the connectome), and the phenome. The phenome comprises the cognitome, symptomatome, and phenomenome (2–4). This framework can be tested and cross-validated using Partial Least Squares (PLS) pathway analysis which combines factor and multiple regression analysis and combines the significant indicator and feature sets into a causal model of schizophrenia. Using this nomothetic network approach, we were able to objectivate the abstract description of schizophrenia and realized a more concrete concept, a phenomenon named “reification of the clinical diagnosis.” Based on the latent variable scores of all feature sets, new categories were exposed using unsupervised pattern recognition methods (2, 3). These novel categories should be cross-validated using supervised learning techniques including soft independent modeling of class analogy (SIMCA) in independent samples. This method is useful to profile phenotype classes (by delineating the features of the novel categories), produce cost-effective classifiers, authenticate or reject class-memberships, and compute the distance between classes, which may help to evaluate quantitative vs. qualitative differences (21, 27).

The above provides a route toward a novel model of schizophrenia based on all features sets. This approach discloses a new network-based definition of schizophrenia phenotypes and pathway classes as well as new treatments of the schizophrenia phenotypes (2, 3, 22). Moreover, the latent variable scores delineate an idiomatic feature profile, which is unique for each patient and may be employed for individualized treatments targeting the most disordered feature sets of the nomothetic model (4).

## Author Contributions

All the contributing authors have participated in the manuscript and approved the final version of the manuscript.

## Conflict of Interest

The authors declare that the research was conducted in the absence of any commercial or financial relationships that could be construed as a potential conflict of interest.
